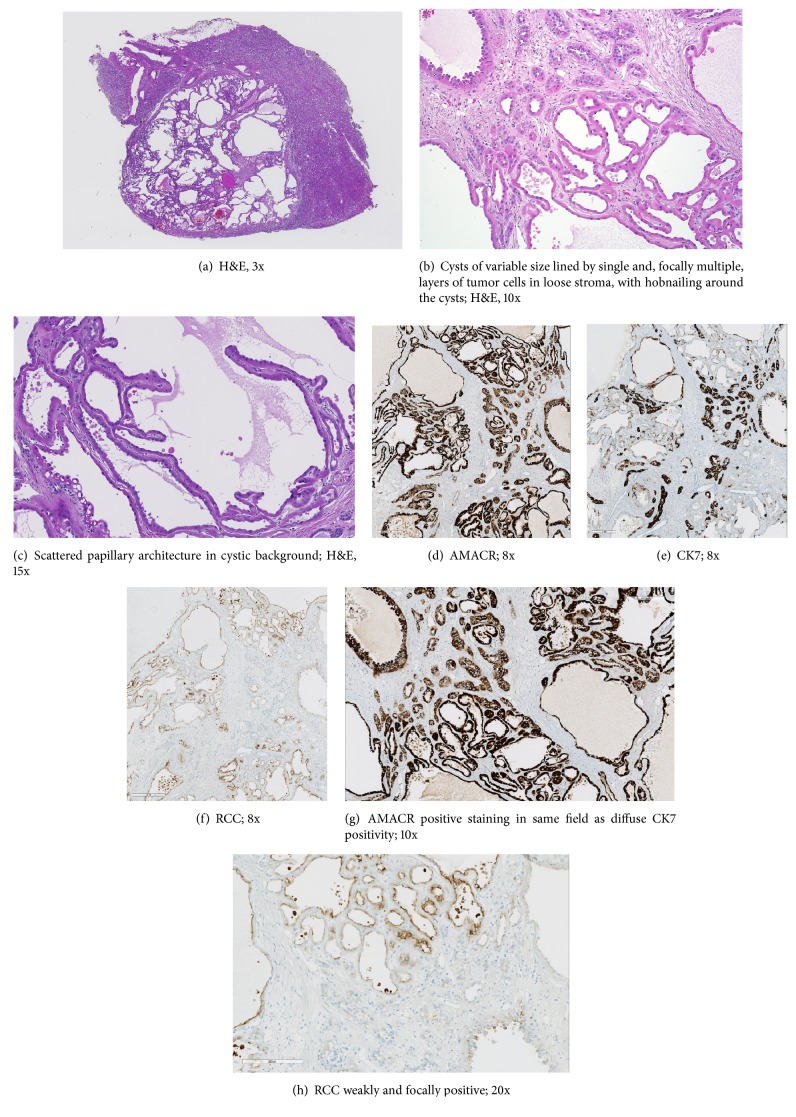# Unusual Suspect: A Case Report of Tubulocystic Renal Cell Carcinoma with Features of Cystic Renal Oncocytoma

**DOI:** 10.1155/2019/2919686

**Published:** 2019-08-05

**Authors:** Jacob D. McFadden, Isabell A. Sesterhenn, Sean Q. Kern

**Affiliations:** ^1^Department of Urology, Walter Reed National Military Medical Center, 8901 Wisconsin Avenue, Bethesda, MD, USA; ^2^Joint Pathology Center, 606 Stephen Sitter Ave, Silver Spring, MD, USA; ^3^Department of Urology, Fort Belvoir Army Medical Center, 9300 DeWitt Loop, Fort Belvoir, VA, USA

## Abstract

Tubulocystic renal cell carcinoma is an uncommon subtype of renal cell carcinoma that was only recently acknowledged by the World Health Organization. There is a relatively small collection of literature dedicated to the features and clinical course of this lesion. Despite its rarity, this diagnosis should remain in the differential for all cystic renal masses. We present a case report of tubulocystic renal cell carcinoma (TC-RCC) with remarkable similarity to cystic renal oncocytoma, highlighting the diagnostic challenges associated with this unusual renal malignancy.

## 1. Introduction

Tubulocystic renal cell carcinoma is a relatively new and unusual variant of renal cell carcinoma that can pose diagnostic challenges. In 2004, Amin et al. first reported a case series in which he described 31 tumors considered “tubulocystic carcinoma,” though the tumor was not included in the World Health Organization classification of Genitourinary Tumors that year [1, 2]. Tubulocystic renal cell carcinoma (TC-RCC) became a distinct entity acknowledged first by the American Joint Committee on Cancer in 2010; it was then included in the Vancouver Classification of Renal Cancer in 2012 and was officially added into the 2016 World Health Organization modified classification of RCC as a newly recognized renal tumor [3, 4].

Characterization of the tumor accelerated after formalizing its classification and has been underscored in earlier literature reviews. The tumor demonstrates a male: female ratio of approximately 7: 1 and a left-sided predominance and is seen in patients in the 5th and 6th decades of life. Unique risk factors for this type of RCC have not been identified. Patients most commonly are asymptomatic on presentation, though hematuria, distention, and abdominal pain are sometimes reported [5]. On imaging, TC-RCC shows a combination of high echogenicity on ultrasound, a cystic or indeterminate appearance on contrast-enhanced CT, and a cystic pattern on MRI; the cysts associated with TC-RCC have been classified from Bosniak I to Bosniak IV [6, 7].

**Figure 1:**
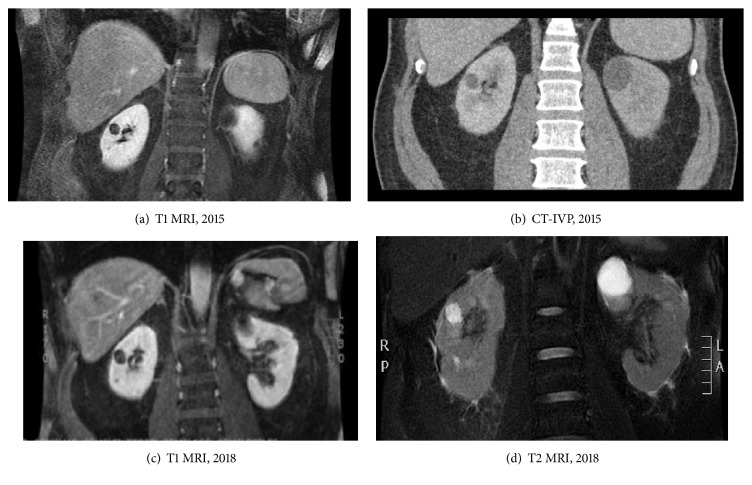


Grossly, the tumors have an average size of 4 cm (with maximum reported size of 17.5cm [8]) and a white-gray color and have variably been described to have a cut surface reminiscent of “bubble wrap,” “sponge,” or “Swiss cheese,” owing to the cystic components of the tumor. Microscopically, the tumor is lined with cuboidal or columnar cells and will often demonstrate hobnail cells in a fibrotic stroma with numerous small tubules and cysts. The tumor cells typically have prominent nucleoli and few mitotic figures [1, 5, 8–10]. TC-RCC tumor cells typically stain positive for CK8, CK18, CK19, parvalbumin, CD10, P504S, AMACR, and vimentin. HMWK and CD117 are typically negative [11].

The differential for cystic masses of the kidney is broad and includes papillary RCC, fumarate hydratase-deficient RCC, collecting duct carcinoma, clear cell renal cell carcinoma with prominent cysts, multilocular cystic renal cell carcinoma, cystic nephroma or mixed epithelial and stromal tumors, synovial sarcoma, and cystic renal oncocytoma, in addition to TC-RCC [8, 12]. Notably, TC-RCC has been found coexisting in tumor specimens that also harbor renal papillary carcinoma and other forms of renal cell carcinoma [13]. Of that differential, cystic renal oncocytoma can be particularly challenging to distinguish from TC-RCC based on its clinical and histopathologic features.

We present a case of tubulocystic renal cell carcinoma with similarity to cystic renal oncocytoma (CRO), in the setting of bilateral cystic disease, highlighting the diagnostic challenges of this relatively new and uncommon condition.

## 2. Case Report

The patient is a 59-year-old male who presented with interval increase in size of an endophytic right interpolar Bosniak III renal cyst on surveillance imaging. The Bosniak III cyst had developed in the setting of mild bilateral cystic disease identified on previous imaging (Figures 1(a)–1(d)). His medical history was notable for hypertension, hyperlipidemia, type II diabetes, and gout, and a baseline GFR of 77 mL/min; he was on appropriate medications for his comorbidities. The patient did not have a family history of genitourinary malignancy, including no family history of renal cancers; there was also no history of skin or uterine leiomyomas. He endorsed a 22-pack-year history of smoking and denied history of toxic environmental exposures. The patient elected to discontinue his active surveillance and proceed with surgical extirpation of the Bosniak III cyst. He was asymptomatic with a normal lab profile at the time of surgery.

He underwent an uncomplicated robot-assisted right partial nephrectomy for his 1.9 × 2.0 × 1.6cm renal mass. He recovered well during the postoperative period.

## 3. Histopathologic Report

The pathologic analysis of his cystic lesion confirmed negative margins. Tissue analysis was remarkable for a well-circumscribed renal mass consisting of cysts, which varied in size from small, more closely packed tubules to larger simple cysts, separated by fibrous stroma, with some areas demonstrating papillary architecture. The cysts were predominantly lined by a single layer and occasionally by multiple layers of cells with variable amounts of eosinophilic cytoplasm resembling oncocytic cells (Figures 2(a)–2(c)). In areas, the tumor cells had a hobnail appearance and focally they contained intracytoplasmic vacuoles. Nucleoli were prominent (grade 3), typical of tubulocystic carcinoma. Ovarian-type stroma seen in cystic nephromas, seen almost exclusively in women, was absent. By immunohistochemistry, the tumor cells were focally positive for CK7 and 34-beta-E12 and diffusely positive for AMACR and vimentin. Fumarate hydratase loss was not detected. The cells were weakly positive for RCC, but negative for S100 protein and c-kit. The proliferative activity in the Ki-67 stain was low (Figures 2(c)–2(g)).

The differential diagnosis generated by the histopathology report included an unusual variant of oncocytoma and the rare tubulocystic renal cell carcinoma. The diagnosis of TC-RCC was supported by the diffuse and strong reaction for vimentin and the lack of CD117.

Given the information collected, the mass was classified as a pT1a renal cell carcinoma. Established National Comprehensive Cancer Network (NCCN) and American Urological Association (AUA) guidelines were used to generate the patient's short-term follow-up plan: a repeat clinic visit 6 months from the date of surgery, with repeat imaging and labs at that time [14].

## 4. Discussion

Oncocytoma is a common, benign renal mass that is well known for its ability to mimic malignant lesions of the kidney, especially on clinical and imaging criteria. The treatment for these lesions is often surgical removal, as they can grow quickly and are difficult to distinguish from renal cell carcinoma on active surveillance. Once removed, the challenge of oncocytoma can persist, as they have well-described histologic similarities to malignant lesions, most notably to eosinophilic chromophobe renal cell carcinoma [15]. Immunohistochemistry is paramount for these cases and can be the separating factor in deciding whether a lesion is benign or malignant.

Approximately 3-7% of renal oncocytomas will demonstrate a tubulocystic histologic pattern [11, 16]. In these situations, the distinction between cystic renal oncocytoma (CRO) and tubulocystic renal cell carcinoma can be nuanced. Skenderi et al. compared the morphologic features and IHC profile of 24 cystic renal oncocytomas and 15 TC-RCCs, noting a handful of key differences. Grossly, CRO will often have more solid components and a less prominent “bubble wrap” appearance. On microscopy, CRO will have tumor islands, unlike TC-RCC. Additionally, TC-RCC shows higher grade nucleoli, mitotic figures, and necrosis—all features not typically observed in CROs [11].

TC-RCC will typically stain negative for CD117, but positive for CD10, AMACR, and CK7. Diffuse vimentin positivity and high Ki-67 proliferative indices (>15%) can also help identify TC-RCC, especially when differentiating from cystic renal oncocytoma [11, 17]. In this case, the lack of CD117 and the strongly positive reaction for vimentin clinched the diagnosis of TC-RCC.

Unlike renal oncocytoma, TC-RCC is uncommon and malignant. Most lesions are small and indolent, though progression and/or metastasis have been reported [5, 8]. Per AUA guidelines, partial nephrectomy is the preferred option for cT1a renal masses in most situations. Because of the paucity of data on TC-RCC, treatment strategies for these advanced cases remain experimental and unproven. Sunitinib and everolimus both have been trialed in the treatment of metastatic TC-RCC [8]. Despite the recent classification of this tumor and its infrequency, strides have been made to define the lesion. Yang et al. used gene expression microarray analysis to demonstrate a unique molecular signature of TC-RCC, as compared with other renal tumors and normal renal tissue. Their data revealed that TC-RCC is closely related to papillary renal cell carcinoma, with further analysis placing it between low- and high-grade papillary RCC. They also discovered that TC-RCC shows trisomy 17 but does not demonstrate trisomy 7, unlike papillary RCC, which typically has both trisomy 7 and 17 [9]. Onsukoya et al. also used gene expression profiles to distinguish TC-RCC from collecting duct carcinoma [18]. A detailed molecular analysis provided by Lawrie et al. demonstrated noncoding RNA and mutational profiles that confirmed a distinct genetic signature for this tumor type. These in-depth analyses have added greatly to the characterization and understanding of this renal cancer subtype.

It is clear that despite an increasing collection of literature, more studies will be required to further characterize TC-RCC and optimize treatment and surveillance strategies for this lesion moving forward. It is unclear whether renal cystic disease is a risk factor for TC-RCC or higher Bosniak scores more frequently harbor TC-RCCs (TC-RCC has been reported in both Bosniak I and II cysts) [6, 8]. In this case report, the distinction between cystic renal oncocytoma and TC-RCC was only made with careful immunohistologic qualification. The radiologic, gross, and histologic features of this lesion can easily be confused with tumors both benign (CRO, cystic nephroma) and aggressively malignant (collecting duct carcinoma), highlighting the diagnostic challenges that can be associated with the uncommon tubulocystic renal cell carcinoma.

## Figures and Tables

**Figure 2:**